# Features of Filler Wire Melting and Transferring in Wire-Arc Additive Manufacturing of Metal Workpieces

**DOI:** 10.3390/ma14175077

**Published:** 2021-09-05

**Authors:** Artem Voropaev, Rudolf Korsmik, Igor Tsibulskiy

**Affiliations:** 1 World-Class Research Center ”Advanced Digital Technologies”, St. Petersburg State Marine Technical University, 3 Lotsmanskaya, 190121 St. Petersburg, Russia; r.korsmik@ltc.ru; 2 Institute of laser and welding technologies, St. Petersburg State Marine Technical University, 3 Lotsmanskaya, 190121 St. Petersburg, Russia; igor@ltc.ru; 3 Institute of Machinery, Materials, and Transport, Peter the Great St. Petersburg Polytechnic University, 29 Polytechnicheskaya, 195251 St. Petersburg, Russia

**Keywords:** wire-arc additive manufacturing (WAAM), non-CNT process, droplet transfer, short circuit process

## Abstract

In this paper, we present the results of a study on droplet transferring with arc space short circuits during wire-arc additive manufacturing (WAAM GMAW). Experiments were conducted on cladding of single beads with variable welding current and voltage parameters. The obtained oscillograms and video recordings were analyzed in order to compare the time parameters of short circuit and arc burning, the average process peak current, as well as the droplets size. Following the experiments conducted, 2.5D objects were built-up to determine the influence of electrode stickout and welding torch travel speed to identify the droplet transferring and formation features. Moreover, the current–voltage characteristics of the arc were investigated with varying WAAM parameters. Process parameters have been determined that make it possible to increase the stability of the formation of the built-up walls, without the use of specialized equipment for forced droplet transfer. In the course of the research, the following conclusions were established: the most stable drop transfer occurs at an arc length of 1.1–1.2 mm, reverse polarity provides the best drop formation result, the stickout of the electrode wire affects the drop transfer process and the quality of the deposited layers. The dependence of the formation of beads on the number of short circuits per unit length is noted.

## 1. Introduction

Currently, wire-arc additive manufacturing is one of the promising methods for obtaining high-precision work-pieces (WAAM) [1]. The method uses an electric arc for multilayer cladding of welding wire. The main advantages of this method are productivity, and low cost of equipment and materials. Currently, there are several varieties of WAAM cultivation, the main ones are based on the technologies of gas arc welding with a tungsten electrode (GTAW) [2] and gas melting arc welding (GMAW) [3]. The latter method is most convenient for use in WAAM, since there is no additional problem of positioning the wire feed.

In contrast to laser cladding by wire, when the most favorable spray transferring of filler metal is observed [4,5], in wire-arc additive manufacturing (WAAM GMAW), the material is transferred into the welding pool in the form of droplets. There are three main transfer modes: droplet, spray, and short circuit [6]. The manufacturing conditions depend mainly on the process energy parameters and gas medium composition.

Spray transferring occurs at high current density. A spray consists of many small droplets that are detached from the electrode by the action of a powerful electrodynamic force caused by high welding current (about 200 A when welding with a 1.1 mm wire) [7]. The droplet transfer process has great stability and no spatter. However, the use of spray transfer is limited only to welding technologies due to high heat input. Under the WAAM process, such transfer negatively affects the cladding productivity due to high residual inter-layer temperature. It is necessary to spend considerable time on cooling the deposited layer [8].

Droplet transfer occurs at lower welding current; however, the formed large drops of molten metal can be deflected and, under various forces, leave the welding zone, creating the process instability [9].

The most favorable transfer occurs when the droplet passes through the arc space short circuit. In this case, the welding current is lower than in other transfer modes, and the arc burning is not constant, which reduces the heat input [10].

Currently, most researchers use the cold metal transfer (CMT) method [11,12,13,14,15,16,17]. It allows to separate the molten droplet by mechanical action on the wire, reducing the process temperature. Fronius welding machines remain almost the only equipment focused on wire-arc additive manufacturing. Understanding the material transfer features in the WAAM process and framing the stability criteria can lead to creating alternative process solutions. For example, Henckell et al. [18] investigated electrode stickout influence on droplet formation for the short circuit mode without the CMT method. They note that an increase in electrode stickout of 10 mm led to a reduction in energy per unit length of 10%. However, an increase in contact tube to work-piece distance leads to an increase in electrical circuit resistance. In this case, the droplet size increases, which can negatively influence the piece formation. Xio et al. [8] investigated the side surface roughness of deposited walls at different process parameters. They derived the ratio of the feeding velocity to the process speed at which the side surface formation was the best. However, the connection with droplet transferring has not been established. Panchenko et al. [10] presented the process of controlled short-circuiting metal transfer at high feeding velocity. The new process reduces heat input by 16% compared to the conventional process. Adebayo et al. [19] report a speed limit in the WAAM process. As welding tool travel speed increases, humping starts to take place. Tang et al. [20] have created an effective model for controlling bead geometry. A deep learning method was used to control the beads. A closed-loop control system based on the bead model and in situ monitoring was developed to adaptively control the bead geometry in real time. This system can control bead geometry and improve forming accuracy.

Thus, the WAAM method without CMT is insufficiently studied. This paper presents the results of a study on the filler material mass transfer during wire-arc additive manufacturing. Experiments were conducted to identify the droplet transferring features.

## 2. Materials and Methods

**Figure 1:**
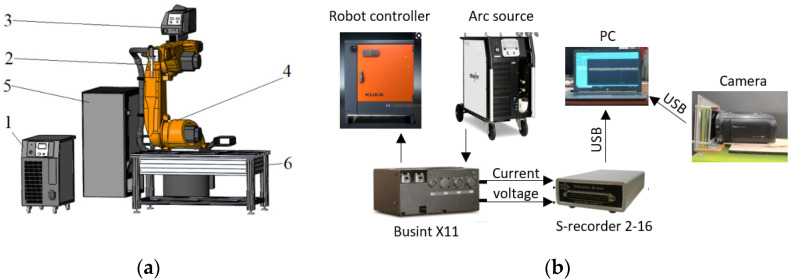
The WAAM station, (**a**)—appearance with components, (**b**)—connection diagram 1—arc source, 2—arc torch, 3—the wire feeder, 4—robot, 5—robot controller, 6—welding table.

The cladding was performed on a WAAM station equipped with a KUKA KR 60 HA robot (Figure 1a) (KUKA Roboter GmbH, Augsburg, Germany). The welding arc source was EWM Alpha Q 551 Expert, the wire feeder—EWM Drive 4X (EWM HighTec Welding GmbH, Mündersbach, Westerwald, Germany). Current–voltage characteristic readings were taken with the Flash Recorder -2-16 signal recorder (signal pickup frequency 10,000 Hz). The connection to the welding circuit was made using the BUSINT X11 interface (Figure 1b). The data from the signal recorder were displayed on the PC. The welding zone was recorded with the Panasonic hc-v770 camera with two optical filters UFS-5 (ultraviolet glass) and ZhS-19 (yellow glass) (GOST 9411-91). The area was illuminated with a diode flashlight.

OK Autrod 12.51 (Elektriska Svetsnings-Aktiebolaget (ESAB), Göteborg, Sweden) wire with a diameter of 1.2 mm was used as a filler and steel 1020 was used as a substrate (Table 1). Pure CO_2_ was used as a shielding gas.

Other parameters of the process: the stickout of the electrode wire was 15 mm, the consumption of shielding gas was 15 l/min.

**Figure 2:**
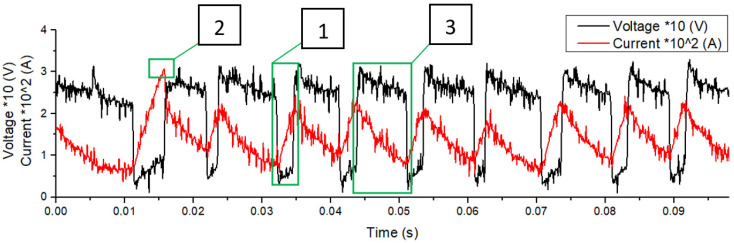
Process oscillogram, 1—short circuit, 2—peak current, 3—arc burning.

The first stage was to evaluate the effect of current polarity on the droplet size, length, and height of the weld pool. Then, a double-factor experiment was performed to evaluate the influence of current and voltage on the drop transfer parameters, and to identify the most optimal deposition mode (parameter range I = 90–160 A, U = 14–26 V). While the wire feed speed was changed from 2 to 4.7 m/min. The number of short circuits, their duration, average peak current, arc burning time, and the diameter of the resulting droplet were estimated by means of oscillogram (Figure 2) and video data. 

In the second stage, experiments on the cladding of 2.5D objects were carried out. During the experiments, the effect of the problem of increasing the departure during the deposition process on the process of droplet transfer through the arc gap and the effect of speed on the stability of the layer-by-layer deposition process were established.

## 3. Results and Discussion

### 3.1. Droplet Transferring

**Figure 3:**
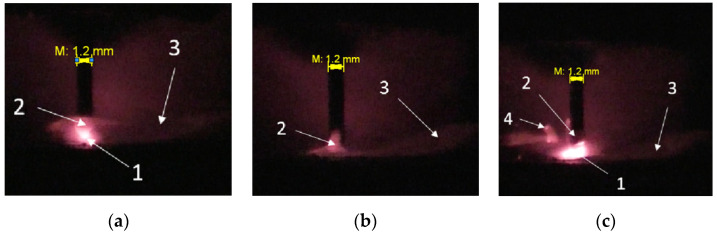
Material transfer result: (**a**)—accumulation of the droplet, (**b**)—shot circuit, (**c**)—the material transfer re-ignition of the arc. 1—arc, 2—droplet, 3—molten pool, 4—spatter.

Filler material transfer mode was obtained during the experiments. The droplet transfer can be divided into two stages. Stage 1—accumulation of molten filler material droplet (Figure 3a). At the beginning of this stage, the welding arc burns and melts the filler wire actively. At the same time, the arc length increases rapidly. As the welding current decreases and the droplet volume increases, the filler wire melting rate decreases. Due to the constant wire feed, the droplet begins to approach the substrate and close the arc space. Stage 2—short circuit (Figure 3b). At the initial stage, under the surface tension force and gravity force, the droplet merges with the pool. The radial component of the electrodynamic force compresses the bridge between the droplet and the wire (pinch effect). The passing current heats up the thinning bridge more and more, which leads to its explosion with spatter formation. After that, the arc is re-ignited, the reactive force pushes the molten metal to the pool end, and the process is repeated (Figure 3c). At the moment of a short circuit, there is a sudden drop in voltage and an increase in current strength.

One of the stability problems in this transfer mode is premature or forced short circuits. That is, the droplet touches the welding pool, and then abruptly pushes it off. In this case, a metal droplet transfers into the welding pool partially, or there is no transfer at all. The nature of random short circuits is related to many factors: the influence of forces controlling the metal transfer, metal evaporation in the places of accidental contact between the droplet and the welding pool, the welding pool and droplet behavior dynamics. Further extensive research is required for a detailed study.

### 3.2. Current Polarity

As it is known, the temperature on the forming droplet surface and the product surface depends on the welding circuit polarity. Pokhodnya et al. [21] declared that droplet temperature is 300–600 °C lower during straight polarity welding (GMAW). Erokhin showed [22] similar results during MMA-welding: temperature of droplet was 200 °C higher when reversed polarity occurred.

**Figure 4:**
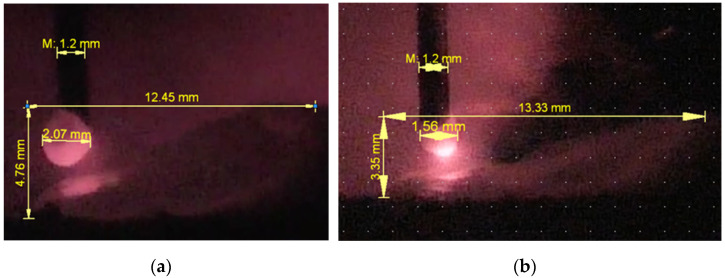
Dimensions of the droplet and the welding pool: (**a**)—straight polarity; (**b**)—reversed.

The surface tension force depends on the molten metal temperature. At the straight polarity, the cathode spot temperature (on the tip of feeding wire) is lower [23,24] as is the temperature of the drop itself, which increases the metal surface tension force (Equation (2)). The droplet stays at the electrode for a longer time and has a larger size, the bridge formation is slowed down due to the greater surface tension force at the straight polarity. The surface tension forces also affect the welding pool height and bead thickness. The bead cladded with greater surface tension on the straight polarity has 4.76 mm in height against bead height 3.35 mm cladded with smaller surface tension on the reversed polarity (Figure 4a,b, respectively). According to the first law of Laplace, for capillary phenomena and under the assumption of spherical droplet surface, the capillary pressure depends on the surface tension coefficient (σ) and the surface tension forces can be determined by the Equation (1) [25]:

(1) $$P_{\sigma }=\frac{2\sigma }{R}{\pi R}_{w}^{2}$$

where σ is the surface tension coefficient (J/mm^2^), depending on the temperature, R is the liquid droplet radius (mm), $R_{w}$ is the electrode radius.

The surface tension decreases almost linearly with the temperature, vanishing at the critical temperature, which is confirmed by the Eötvös Equation (2):

(2) $$\sigma =2.1\frac{\left(T_{\mathrm{crit}}-T\right)}{{\left(M\cdot \frac{1}{\gamma }\right)}^{2/3}}$$

where T is the temperature at the moment (K); $T_{\mathrm{crit}}$ is the metal critical temperature (K), equal to the Gulberg rule [25] $T_{\mathrm{crit}}=\frac{3}{2}T_{\mathrm{boil}}$; M is the molecular weight; $T_{\mathrm{boil}}$ is the boiling temperature (K); γ is the specific weight.

**Figure 5:**
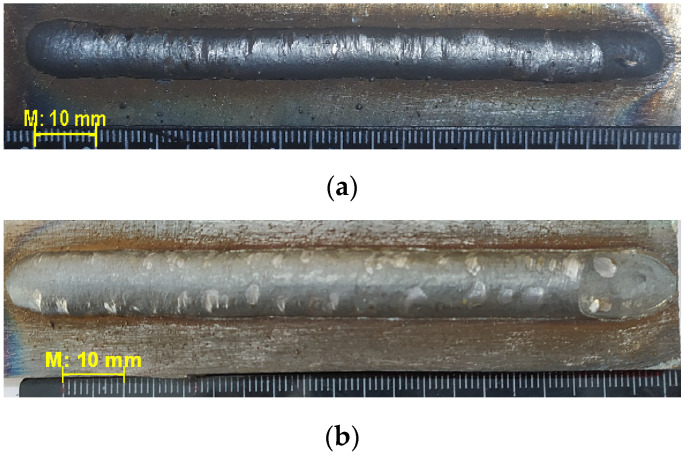
Single beads: (**a**)—straight polarity, average width 7.97 ± 0. 16 mm; (**b**)—reversed, average width 11 ± 0.3 mm.

Figure 5 shows influence of the polarity on bead’s width. The beads on straight polarity have narrower geometry against wider beads on reversed polarity (Figure 5a,b, respectively). The bead deposited at the straight polarity has a height-to-width ratio of 0.59 (Figure 5a). At the reversed polarity the ratio is 0.30 (Figure 5b). When the multilayer cladding with overlaps in each layer is produced, higher height-to-width ratio can lead to defects in the form of lack of fusion between the adjacent layers [26].

Thus, the current polarity influences the droplet formation. The most rapid transition of the molten droplet into the weld pool occurs with reverse polarity. In this case, the geometric parameters of the deposited layers are optimal. All further experiments were conducted at this mode.

### 3.3. Influence of Energy Parameters

**Figure 6:**
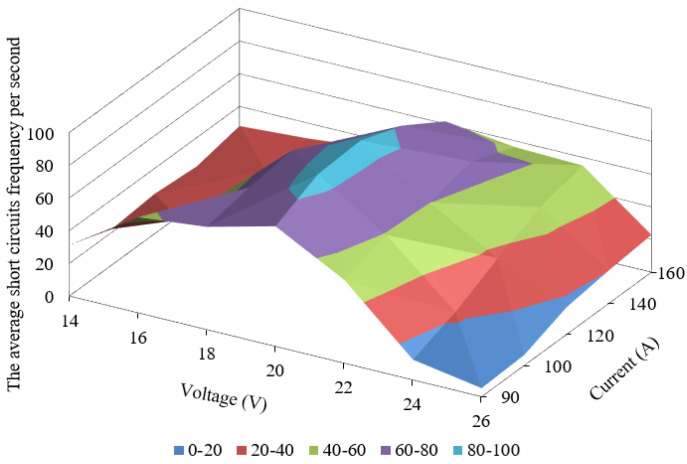
Graph of the dependence of the average short circuit frequency per second on current and voltage at the constant tool travel speed.

Following the experiment results, we obtained a three-dimensional graph showing the dependence of the average short circuits frequency per second on current and voltage (Figure 6).

Analysis of obtained oscillogram and video files were made to explain appearance of extremum in point with parameters I = 120 A and U = 20 V. Modes received at U = 20 V with varying current (Figure 4, Table 2 ) and I = 120 with varying voltage (Figure 5, Table 3) were selected as an object of research.

**Figure 7:**
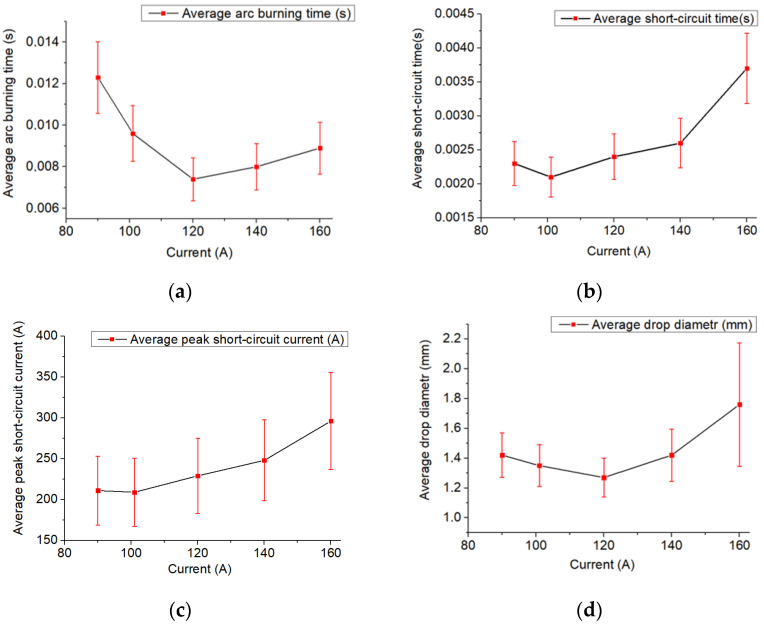
Graphs: (**a**)—average arc burning time, (**b**)—average short circuit time, (**c**)—average peak short circuit current, (**d**)—average droplet diameter dependance on the set current parameters.

As the welding current increases, the arc burning time and the forming droplet diameter decrease. The shortest arc burning time is recorded at the welding current of 120 A (Figure 7a). In this case, there is an increase in the short-circuit time and the average peak arc current (Figure 7b,c), which is related to an increase in the thickness of the bridge between the droplet and the wire. At the welding current of 140 A, the arc space column height has a small value (Table 2), which increases the arc pressure. The droplet is often displaced onto the filler wire side surface, thus increasing the burning time and the droplet diameter (Figure 7a,d). The short-circuit time and the average peak short-circuit current also increase (Figure 7b,c). This process is most sensitive to external disturbances. A further increase in the welding current leads to mechanical contact of the wire with the unformed droplet and the substrate, which further increases the average short-circuit time and the average peak current (Figure 7b,c). During the short circuit, the wire section in contact with the droplet is overheated. The overheated section either completely transfers into a droplet, increasing its size and arc length, and consequently the average burning time, or under the influence of the constant feeding velocity, the heated wire section breaks. The material transferring process is unstable.

The proposed results were achieved by analyzing video clips of the cladding process (Table 2). The arc gap was measured at the end of the short-circuit phase and the occurrence of the welding arc at the start of the droplet accumulation stage.

**Figure 8:**
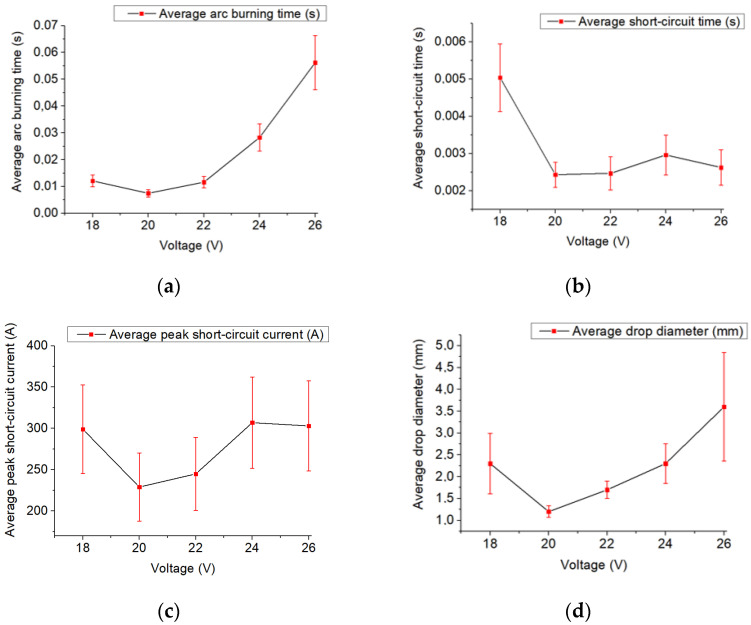
Graphs: (**a**)—average arc burning time, (**b**)—average short circuit time, (**c**)—average peak short circuit current, (**d**)—average droplet diameter dependance on the set voltage parameter.

A similar process is observed as the welding voltage changes (Figure 8). As the welding voltage increases, the arc length (Table 3) and the droplet size increase (Figure 8d). Deviations from the vertical axis and large droplets deformation are observed. In this case, there is an increase in the average peak short-circuit current required for the droplet separation (Figure 8c) without a large increase in the short-circuit time (Figure 8b). At the low voltage, the same process occurs as at the high welding current, which is described above (Figure 7 and Figure 8). At the voltage below 18 V no formation occurred.

**Figure 9:**
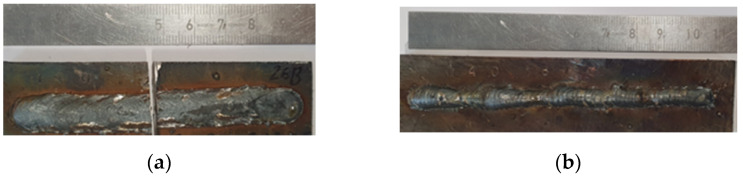
The appearance of the bead, (**a**)—I = 120 A, U = 26 V; (**b**)—I = 120 A, U = 18 V.

Thus, the arc column height plays an important role in the cladding. As it increases, the droplets size increases and the frequency of their separation decreases, the bead becomes uneven in height and width (Figure 9a). As the arc size decreases, the wire and the workpiece come into mechanical contact, which significantly affects the cladding stability (Figure 9b).

Good results of arc characteristics were obtained using U = 20 V and I = 101–120 A. However, for further studies the modes I = 120 A and U = 20 V were chosen due to more value of feeding material and thus better filling ability of bead shape through its length.

### 3.4. Electrode Stickout Influence

**Figure 10:**
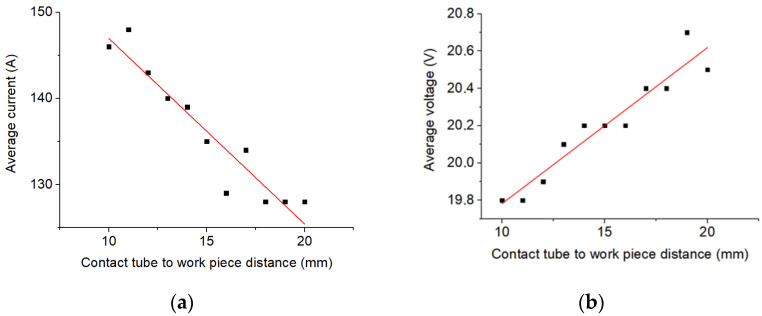
Change in the average cladding current and voltage depending on the electrode stickout, mode I = 120 A, U = 20 V; (**a**)—average current, (**b**)—average voltage.

As the electrode stickout changes, the process average current and voltage values change (Figure 10). As the electrode stickout increases, welding circuit resistance increases [18]. Thereby, welding current drops and voltage increases. This process problems are described above.

**Figure 11:**
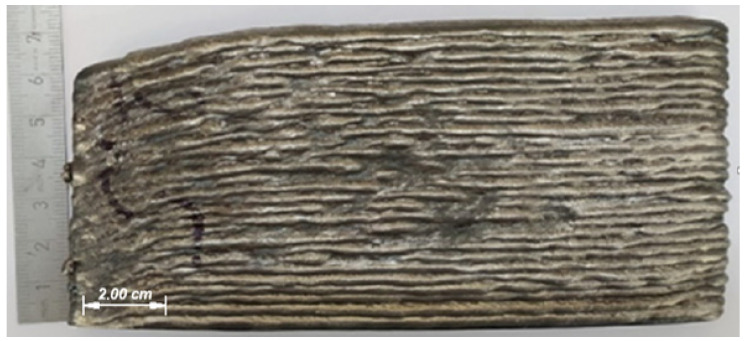
The wall appearance, mode I = 120 A, U = 20 V.

Figure 11 shows the appearance of a wall with 30 layers. The stickout was not adjusted for the first 20 layers, thus, the distance from the current-collecting tip and the workpiece increased from the set 15 mm to 20 mm. Following the adjustment, 10 more layers were deposited, resulting in stickout change up to 17 mm.

As the wall height increases, the stability of the deposited layers side surface formation deteriorates. Therefore, the transfer process changes when the electrode wire stickout length increases. Data in Table 4 show the drop enlargement with increase of the electrode wire stickout. Drop transfer mode change affects the lateral surface of depositing sample in the form of dips and bursts (Figure 11).

Some researchers [27,28] have found that a large droplet size increases the width of the bead by increasing the volume of the weld pool. The height of ripples and their period also increase. The arc burning and short circuit times become longer during transition to the droplets of bigger size. When voltage rises and current falls an increase of the droplet diameter is observed, as well as its average value deviation increases (Figure 7d and Figure 8d). At this moment, the welding pool behavior begins to differ in comparison with the first layers. An excess of metal caused by the large droplets transition is supplanted to the lateral surface, forming overlaps. In turn, in places with a lack of material, unfilled areas are formed. There is the intense ripples on the top surface of the bead. In conditions of layer-by-layer manufacturing, this phenomenon is cumulative. To improve the formation stability, strict control of the electrode stickout is required.

### 3.5. Cladding Speed Influence

**Figure 12:**
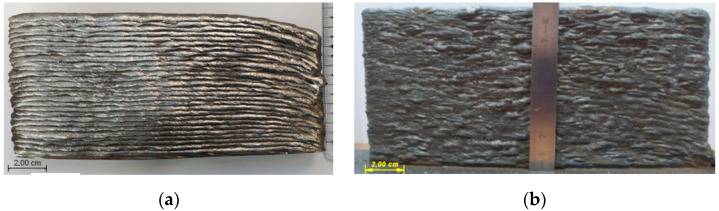
Appearance of the walls made in the mode I = 120 A U = 20 V, the feeding velocity is 3.2 m/min, the tool travel speed: (**a**)—0.12 m/min, (**b**)—0.24 m/min.

The process speed has a major effect on the deposited layers formation stability. During depositing the 30-layer walls it has been found that the process stability strongly depends on the number of short circuits (or drops) per unit length (Figure 12).

In this case, the number of short circuits per 1 mm of length was equal to 50 and 25 (Figure 12a,b, respectively). As can be seen the greater amount of feeding droplets leads to stable formation (Figure 12a).

It is noted that the more drops transfer to the weld pool per length unit, the smoother the surface is formed [27,29]. At a lower cladding speed, the solidification rate of the weld metal is lower. The drop transition impulse tends to be damped due to the larger weld pool at a lower welding speed [27]. As a result, the metal remains in a liquid state for longer [30]. This allows it to be evenly distributed over the entire deposited area.

## 4. Conclusions

1. The arc column height plays a key role for deposition, ensuring a stable transfer of the filler material through the arc space. It is influenced by the main process parameters: welding current and filler wire feed, voltage. Selecting optimal parameters allows us to obtain minimal welding arc fluctuations during cladding, and consequently, better formation of deposited layers. The optimal transfer is achieved at an arc height of 1.1–1.2 mm for wires with a diameter of 1.2 mm.
2. The most stable filler material transfer is achieved at the reversed polarity.
3. In the course of cladding, electrode stickout strict control is required to achieve stable formation of deposited layers. In this regard, it is required to take measures to select the optimal layer increment in order to prevent an increase in the electrode stickout, or use special triangulating sensors to control the welding tip height during pauses between layers. Feedback systems will prevent changes in the drop transfer by continuous adjustment of the electrode wire stickout length which positively affects the stability of the surfacing process.
4. It is noted that the number of short circuits per track unit can affect the stability of the formation of deposited layers.

During the experiments, the repeatability of the results obtained was confirmed. Re-analysis of the first bead of 2.5D objects confirmed the data obtained when surfacing single beads.

The obtained data can form the basis for the development of technology without the use of special technologies for controlling the material transfer, as well as for creating systems with feedback.

## Figures and Tables

**Table 1:** Substrate and wire (OK Autrod 12.51) chemical composition.

Components	Fe (%)	C (%)	S (%)	P (%)	Si (%)	Mn (%)	Cr (%)	Cu (%)	Ni (%)	As (%)
substrate	Balance	0.17–0.24	Up to 0.04	Up to 0.035	0.17–0.37	0.35–0.65	Up to 0.25	Up to 0.3	-	0.08
wire	Balance	0.1	Up to 0.025	Up to 0.03	0.75	1.5	Up to 0.3	Up to 0.3	Up to 0.3	-

**Table 2:** The welding current influence on the arc height at U = 20 V.

Process Parameters I (A)	Average Arc Column Height (mm)
90	1.5
101	1.23
122	1.11
140	0.85
160	1.19

**Table 3:** The welding voltage influence on the height at I = 120 A.

Process Parameters U (V)	Average Arc Column Height (mm)
18	2.21
20	1.11
22	1.5
24	1.84
26	2.9

**Table 4:** Cladding characteristics at the 1st and the 20th layers.

Layer Number	Number of Short Circuits	Average Short Circuits Time (s)	Average Arc Burning Time (s)
The 1st layer	100	0.0024	0.0075
The 20th layer	63	0.0037	0.012

## Data Availability

The data presented in this study are available on request from the corresponding author.

## Abbreviations

WAAM—wire arc additive manufacturing, GTAW—gas tungsten arc welding, GMAW—gas melting arc welding, CMT—cold metal transfer, MMA—manual metal arc welding.
